# The use of primary murine fibroblasts to ascertain if *Spirocerca lupi* secretory/excretory protein products are mitogenic ex vivo

**DOI:** 10.1186/s12917-017-1162-9

**Published:** 2017-08-22

**Authors:** Kgomotso Sako, Ilse jv Rensburg, Sarah Clift, Vinny Naidoo

**Affiliations:** 1 Section of Pharmacology and Toxicology, Department of Paraclinical Sciences, Faculty of Veterinary Science, University of Pretoria, Private Bag X04, Onderstepoort, 0110 South Africa; 2 University of Pretoria Biomedical Research Centre, Faculty of Veterinary Science, Private Bag X04, Onderstepoort, 0110 South Africa; 3 Section of Pathology, Department of Paraclinical Sciences, Faculty of Veterinary Science, University of Pretoria, Private Bag X04, Onderstepoort, 0110 South Africa

**Keywords:** *Spirocerca lupi*, Ex vivo culture, Viability, Mitogenic, Murine fibroblasts

## Abstract

**Background:**

*Spirocerca lupi* is a nematode that parasitizes vertebrates in particular canids, by forming nodules in the thoracic cavity specifically in the oesophagus. In 25% of *Spirocerca* infections of the domestic dog, nodules progress from inflammatory to pre-neoplastic to sarcomatous neoplasia. With the mechanism of neoplastic transformation being incompletely understood, this study investigates if *S. lupi* parasite proteinaceous secretory/excretory products (ESPs) play a role in the neoplastic transformation.

**Methods:**

To facilitate collection of ESPs, we maintained naturally harvested adult parasites in the laboratory under artificial conditions. Media in which the parasites were grown was subsequently evaluated for the presence of proteinaceous compounds using a mass spectroscopy library as well as for their ability to be mitogenic in primary murine fibroblastic cells.

**Results:**

Chromatrography of the ethyl acetate extracted incubation media showed the presence of 9 protein compounds, of which three were identified as non-specific proteins isolated from *Nematostella vectensis, Caenorhabditis brenneri* and *Sus scrofa*, with the rest being unknown. Acetone, methanol, hexane and ethylacetate extracted culture media were unable to induce a mitogenic change in primary murine fibroblasts in comparison to the controls.

**Conclusion:**

While no mitogenic effect was evident, further studies are required to understand the role of worm excretory/secretory products on clastogenesis under chronic exposure. In addition, while not of primary importance for this study, the observed duration of parasite survival indicates that ex vivo studies on *S. lupi* are possible. For the latter we believe that the worm culture method can be further optimized if longer survival times are required.

## Background

*Spirocerca lupi* is a nematode parasite with higher prevalence in both tropical and subtropical countries, despite its global distribution [1]. The life cycle of the parasite is well understood and includes intermediate hosts (coprophagous beetles), paratenic hosts (wild birds, lizards, rodents, hedgehogs, rabbits and poultry) and the definitive host (dogs). *S. lupi* eggs containing stage 1 larvae (L1) are shed in the faeces or with the vomit of the canine definitive host. Coprophagous beetles of the Scarabaeidae family feed on the vomit or faeces, ingesting the larvated eggs. The emerging larvae (L1) then encyst in the tissues of the beetles and develop to stage 3 larvae (L3) within approximately 2 months. Paratenic hosts or the definitive host feeds on the coprophagous beetles, ingesting the L3 larvae. Following ingestion of L3 larvae by the definitive host, the larvae get released in the gastric lumen, then migrate within the gastric mucosa, serosa and gastric/coeliac arteries, reaching the caudal thoracic aorta, where they develop to stage 4 larvae (L4) and then to young adults. Thereafter, they migrate to the caudal thoracic oesophagus, where they settle and form nodule(s) in the serosa and submucosa [1–3].

Once a dog is infected with, the clinical signs most often seen include regurgitation, weight loss and/or dysphagia [4, 5]. The pathognomonic diagnostic lesions for spirocercosis include scarring of the caudal thoracic aorta with osseous metaplasia and/or aneurysm formation, caudal thoracic ventral vertebral body spondylitis and the formation of nodule(s) in the caudal thoracic esophagus [4]. In up to 25% of *S. lupi*-infested dogs, the esophageal nodules progress from inflammatory esophageal nodules to pre-neoplastic fibroblastic nodules and eventually to sarcoma [6]. *S.lupi*-induced sarcomatous neoplasia has been further classified histologically as osteosarcoma (the predominant *S. lupi*-induced sarcoma), fibrosarcoma and anaplastic (undifferentiated) sarcoma [2].

While the progression of the *S. lupi* nodule to neoplasia is well documented and described, the mechanism underlying the progression to neoplasia is poorly understood with current hypotheses linking neoplastic transformation to chronic inflammation [3, 7–9]. According to this supposition, it is believed that chronic irritation induces cellular metaplasia which eventually results in neoplastic transformation. In support of this, various studies have demonstrated that the initial esophageal inflammatory lesion induced by the parasites and its subsequent progression to neoplasia is associated with changes in C-reactive protein, vascular endothelial growth factor (VEGF), fibroblast growth factor (FGF), platelet-derived growth factor (PDGF) and interleukin 8 concentrations [3, 7–9]. However, these studies did not conclusively demonstrate that the inflammation seen was the cause of the progression to neoplasia, as opposed to being the expected response to the presence of a parasite.

Whilst inflammation is known to underpin carcinogenesis in some cases, very little attention has been given to the possibility that the worm could be secreting/excreting a mitogenic substance that might be responsible for the nodule formation and later neoplastic change. The human medical literature, to date, has reported numerous cases of infectious agents inciting direct neoplastic change. *Clonorchis sinensis*, a liver fluke, induces clonorchiosis and subsequent cholangiocarcinoma, and has been found to cause an increase in cyclins E and B, which play an important role in the regulation of the cell cycle, in the induction of mitosis, and in the behavior of neoplastic cells [10]. *Opisthorchis viverrini*, another liver fluke, known to induce cholangiocarcinoma in humans is also suspected to induce oncogenesis via changes in the release of cyclin proteins [11]. As a result we believe it is important to evaluate the environment surrounding the parasite for the presence of oncogenic protein substances. The following study attempts to evaluate the secretory/excretory protein products (ESPs) of the *Spirocerca* adult worm collected from an ex vivo environment for their mitogenic effect using murine fibroblasts. In addition, since S*. lupi* worms have never been maintained in the laboratory to our knowledge, the other aim of this study was to establish if it was possible to maintain the parasite under laboratory conditions for ex vivo investigation, even if for limited periods.

## Methods

### Collection and maintenance of worms

Adult worms were collected from domestic dogs (*n* = 4) as soon as possible following euthanasia, and in all cases collection was opportunistic following euthanasia on humane grounds. Harvesting was approved by the Animal Use and Care Committee of the University of Pretoria (V063/12). Following esophageal nodule incision and careful removal of adult worms, they were placed into pre-warmed saline, Iscove’s Modified Dulbecco’s Medium (Iscove’s), Dulbecco’s Modified Eagle’s Medium (DMEM), Ham’s F12 Medium (Ham’s) or Roswell Park Memorial Institute (RPMI) 1640 medium (RPMI) prior to transportation to the laboratory. All media was purchased from Highveld Biologicals (South Africa) and contained phenol red as a pH indicator. Prior to plating, parasites were rinsed three times using sterile phosphate buffered saline (PBS) to remove the detritus on the parasite’s cuticle. The parasites were thereafter immersed in 2 ml of one of the four mentioned media or in pre-warmed saline (*n* = 6 for RPMI and *n* = 9 for all other media) under serum free conditions. Plates were maintained in a humidified environment of 5% CO_2_ in oxygen (Carbogen) at 37 °C. Culture media or saline was replaced at 48 h intervals. Following every media change, the removed media was frozen at −80 °C for future use. Worm survival (viability) was ascertained by parasite motility, the integrity of the esophagus and the color of the cuticle when non-motile.

### Liquid chromatography

The supernatant (culture media or saline) was concentrated by removing the water using a Lyoquest freeze dryer (Telstar®) at a temperature of −77 °C and vacuum of 0.046 m-bar. Thereafter, samples were diluted in a mixture of water and acetonitrile (1:1). At least 20 μl of each of the samples was injected into an ultimate 3000 ultra-high performance liquid chromatography (U-HPLC) (Thermo Scientific and Dionex) fitted with an Acclaim™ 120 C18 column (Dionex) with a particle size of 3 μm, 2.1 mm × 100 mm diameter and average pore diameter of 120 Å. The mobile phase (0.1% formic acid and water, 0.1% formic acid and acetonitrile) containing formic acid as an ion pairing agent was added to the column at a flow-rate of 0.3 ml/min. The U-HPLC was connected to a MicroTOF-QII (Bruker™) high resolution mass spectrophotometer (MS). Peaks were identified by use of an attached library.

### Mitogenic assay

#### Preparation of the supernatant

Frozen media collected during the first incubation was thawed and mixed 1:1 with acetone, hexane or methanol. Mixed samples were subsequently centrifuged at 3500 rpm for 3 min (Allegra®, Beckman Coulter), at room temperature. Samples were rapidly frozen in a dry ice/methanol mixture to potentially remove the aqueous phase. After the ice-bath, the supernatant was dried using pressurized nitrogen (15 psi) at 60 °C for 60 min. The dried samples were preserved at 4 °C in a fridge until assessed. Prior to use the extracts were reconstituted at 750ul of their respective extraction solvent to 250ul of media, 500ul of solvent to 500ul of media or 250ul of solvent to 750ul of media.

#### Establishment of cell cultures

Skin samples were harvested from the pinnae of Balb/c mice (either sex ±3 months old) supplied by the UPBRC (University of Pretoria, Biomedical Research Centre). Sample collection was purely opportunistic following scheduled terminations from another approved study. Samples were cleaned using Bioscrub (Chlorhexidine gluconate), sterile water and 70% alcohol, digested in pre-warmed collagenase and hyaluronidase (Sigma Aldrich) solution at 37 °C in a humidified 5% CO_2_ incubator for 24 h. After digestion, samples were centrifuged at 42 g, at 500 rpm for 5 min and the supernatant discarded. Pellets were suspended in DMEM HI (Sigma) and 40% heat inactivated fetal bovine serum (FBS-Scientific group) growth medium and gently agitated using the serological pipette tip and transferred into 50 ml TC flasks (Nunc, Thermo ScientificTM) with 5% antibiotic (Streptomycin, Neomycin and Penicillin). Cultures were maintained at 37 °C in 5% CO_2_ in growth media for a week. After the first week of incubation, the culture medium was replaced with 10% FBS in DMEM in TC flasks and these were changed twice a week. Once cultures reached confluence of approximately 80–90%, they were rinsed twice with trypsin-versene solution and trypsinized. Cells were counted using the trypan blue exclusion method.

### Exposure studies

The extracted Iscove’s media were filter-sterilized (0.25 μm filters, Millipore corp.). Fibroblasts in suspension (400 μl) were dispensed into each well of 8-well chambered slides (Lab-Tek, USA) and incubated for 24 h. After incubation, the culture medium was changed and fibroblasts were treated with 40 μl of one of the three concentrations of solvent-extracted secretory/excretory product for 48 h (final extract exposure of 10, 20 and 30 μl before dilution in the media was taken into consideration). The experiment was carried out in triplicates and in all cases compared to their respective solvent control. After the prerequisite exposure period, the slides were air dried, fixed in 100% methanol, and stained with Lily Mayer’s haematoxylin and eosin using a laboratory optimized method. Detailed cytological evaluation was performed on digital photographs (4140 × 3096 pixel) obtained using an Olympus DP72 digital camera attached to an Olympus light microscope (Olympus, Japan). The photographs were downloaded onto a computer and adjusted to 50% (1360 × 1024 pixels) of their initial size in Paint (Microsoft Windows 7). A grid was superimposed over the photographs. Fibroblasts (*n* = 100), both normal and mitotic, were counted in an anticlockwise manner for each photograph.

### Establishment of the cause of contamination

Prior to culture, the pH of thawed culture media or saline was measured using a standard pH meter. Samples were plated on MacConkey agar, sheep’s blood agar (BAP), colistin-nalidixic acid agar (CNA) and broth containing methyl-umbellinferyl-β-glucuronide (MUG) and incubated at 35 °C in an aerobic atmosphere in an incubator for 24 h. Thereafter, growth of colonies and lactose consumption in samples plated on MacConkey agar was monitored. The recovered colonies of BAP and CNA were tested for biochemical reaction using catalase (−), lancefield group D (+) and pyrrolidonyl arylamidase [12].

## Results

**Fig. 1:**
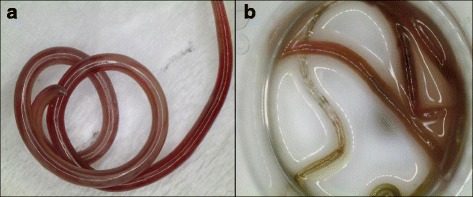
Healthy adult *Spirocerca lupi* worm (**a**) and a dead parasite (**b**). The healthy worm is pink in colour while the dead worm has lost it colour

At the time of harvesting from the nodules the worms were curled-up and showed resistance to gentle pulling force. On placement in the warmed media the worms showed variable movement with time to harvest appearing to affect motility i.e. worms collected immediately after euthanasia of the host appeared to be more active and retained their pink-colored cuticle (Fig. 1a). After approximately 30 min within the warmed culture media, worms once again showed movement. Irrespective of the culture media, the worms all died by 96 h after harvest (Fig. 1b). Viability was longer in the plain saline, with only half of the parasites dying by 96 h, with the rest surviving to 144 h.

In all cases the death of the worms were associated with change in colour of the phenol red to yellow and an unpleasant smell of the media, with concurrent pH change of 7.38 ± 0.45; 7.67 ± 0.14; 7.24 ± 0.51 and 7.53 ± 0.26 for the Iscove’s, DMEM, Ham’s, RPMI media from a pH of 7.4. The pH of the saline control remained fairly constant at 6.00 ± 1.09 and remained unchanged from the pre-treatment pH of 6. Culture of the media and saline both revealed the presence of *Escherichia coli, Enterococcus faecalis, Pseudomonas aeruginosa* and *Serratia marcescens*.

**Fig. 2:**
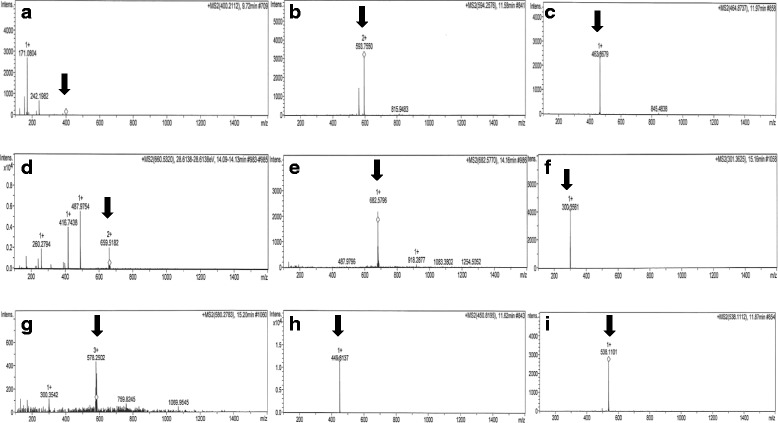
Proteins separated on LC-MSMS with mass to charge ratios of 400.2112 m/z (**a**); 594.2576 m/z (**b**); 464.8737 m/z (**c**); 660. 5320 m/z (**d**); 682.5770 m/z (**e**); 301.3625 m/z (**f**); 580.2783 m/z (**g**); 450.8195 m/z (**h**); 538.1112 m/z (**i**)

Liquid chromatography revealed the presence of nine peaks, indicating the existence of nine different protein compounds of various sizes with mass to charge ratios of 166.0868 to 928.4730 m/z (Fig. 2) in the Iscoves and RPMI media. No peaks were present in the other media or the saline. Despite nine peaks being present, comparison with the molecular ion masses published from the protein library allowed for the characterization of only three of these proteins (same as the non-specific proteins produced by *Nematostella vectensis,* Caebren (*Caenorhabditis brenneri*) and *Sus scrofa*, with no information being available on their potential carcinogenic effects. None of the other chemicals were identifiable.

**Fig. 3:**
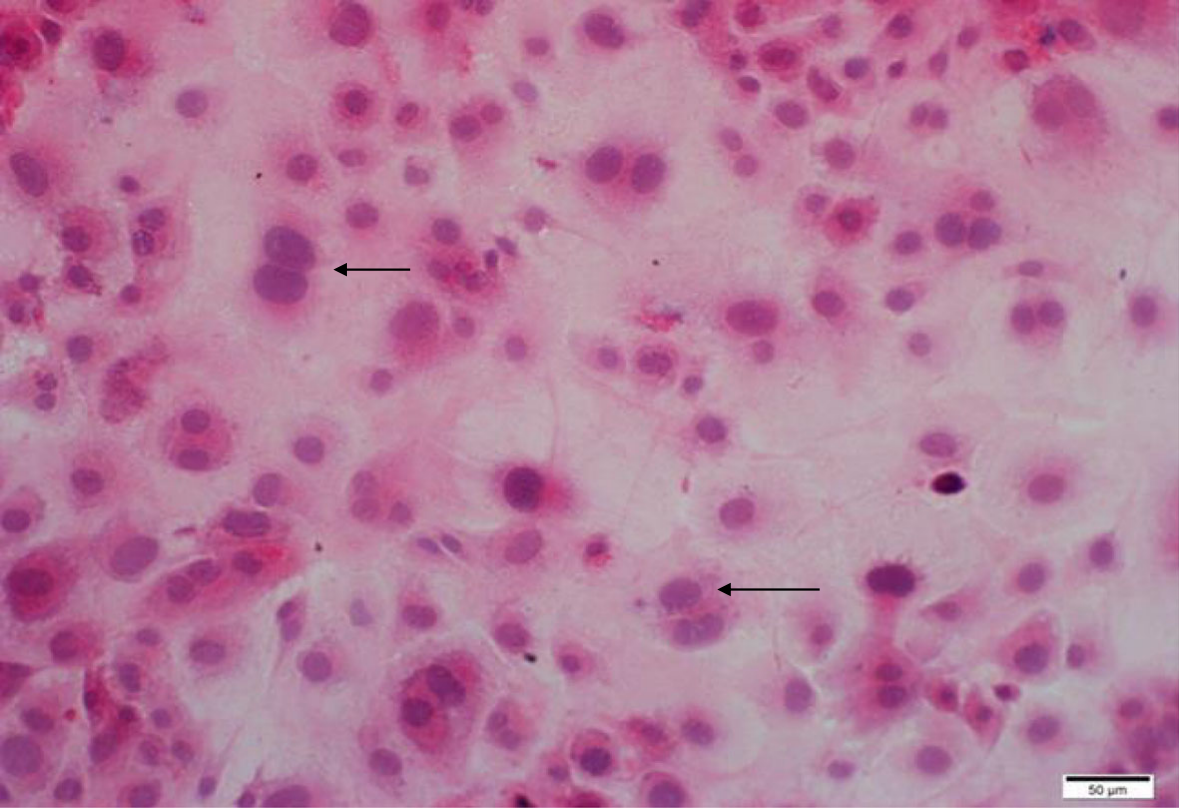
Photograph of fibroblasts harvested from Balb/c mice, stained with HE, showing evidence of nuclear pleomorphism (bi-nucleates and karyomegaly) (arrows). Sample obtained from the acetone treated slides

**Fig. 4:**
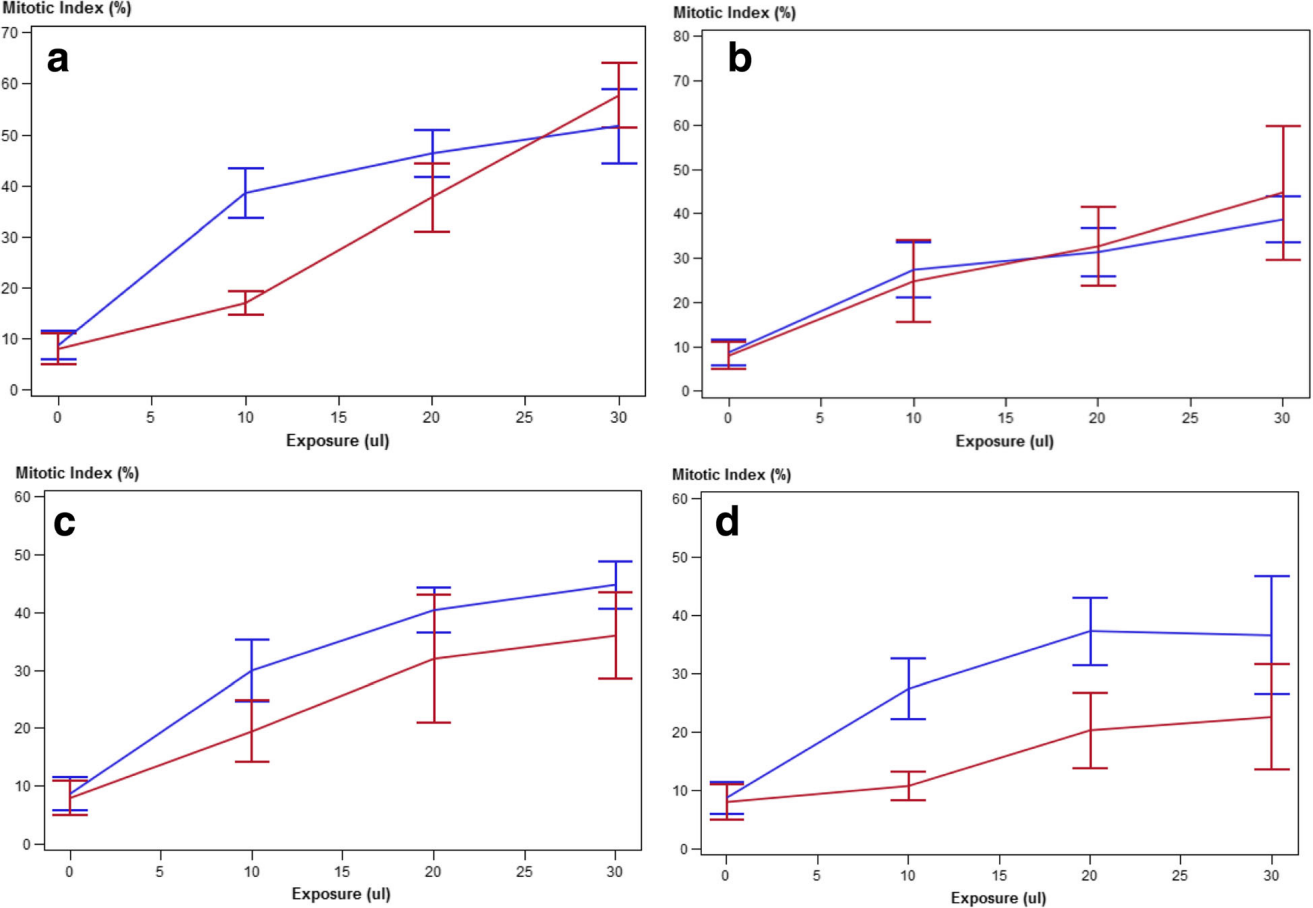
The effect of adult *S. lupi* extracts dissolved in acetone (**a**); hexane (**b**); methanol (**c**); ethylacetate (**d**) compared to the effect of the solvent control (blue) on murine fibroblasts

Following the exposure of fibroblasts to adult *S. lupi* ESPs (at various dilutions, 10, 20 and 30 μl), HE-stained cytological preparations were assessed for an increase in mitotic rate which would have been indicative of clastogenesis (Fig. 3). An increase in fibroblast proliferation was evident in both the adult *S. lupi* ESPs extracts and in the organic solvent groups, with a clear concentration-response relationship for the latter (Fig. 4). Based on the similar results, it was evident that the *S. lupi* ESPs had no additional mitogenic effect over and above that of the solvents alone under experimental conditions.

## Discussion

The aim of this study was to ascertain if the *S. lupi* adult parasite changed its surrounding environment through the release of secretory/excretory protein products, and whether any of these products could be the reason for the parasites ability to induce carcinogenic changes in the host. The latter was undertaken by placing live adult worms into specific media/saline for a limited period. Only the Iscoves’s and RPMI media showed a difference in their protein content in comparison to the said media prior to culture. Of the two, the Iscove’s media was selected for further analysis for the presence of a mitogenic effect, as preliminary TLC separation demonstrated the most intense staining bands (results not shown). In all cases the proteins identified were above 160 m/z. While in total nine additional proteins were identified on the spectra through LC-MSMS, only three of these were previously identified from other organisms. The three proteins have, however, thus far not been subjected to any further testing for the purpose of this study, nor could any published studies on the function or significance of these proteins be found. Another important question that should be answered is whether these proteins were from parasite or bacterial origin.

In an attempt to establish if these proteins could be mitogenic a cell culture assay was used. Following the exposure of fibroblasts to adult *S. lupi* ESPs (at various dilutions, 10, 20 and 30 μl), the in vitro fibroblasts were investigated in cytological preparation for an increase in mitotic rate, which would have been indicative of clastogenesis. For the exposure, murine fibroblasts were harvested and exposed to extracts of excretory/secretory substances for a period of 48 h. The principle of the assay was based on use of primary fibroblast cultures to ascertain the mitogenic effect of environmental chemicals, under in vitro conditions [13]. As an increase in fibroblast proliferation was evident in both the adult *S. lupi* ESPs extracts and in the organic solvent groups, we concluded that *S. lupi* ESPs had no additional mitogenic effect over and above that of the solvents alone under the conditions of the study design. While the absence of a mitogenic effect tends to suggest that the parasite is not inducing its effect via the release of secretory/excretory proteinaceous substances, the time progression of infection to tumour development in vivo still needs to be taken into consideration. As such we believe that to conclusively rule out the effect of potentially mitogenic compounds, it may be necessary to undertake repeated exposure of the primary fibroblast cultures, in such a manner that with the periodical media change, the excretory/secretory products are replenished over a longer period of time.

While the main objective of the study was to ascertain if the parasites were releasing potentially mitogenic secretory/excretory products, to achieve this the parasite had to be maintained outside of its host for a period of time. To our knowledge this is the first description of an attempt to maintain adult *S. lupi* worms alive outside of the host animal, even for the short periods required for this study, and thus warrants some discussion. The worms in question were collected opportunistically from recently euthanized dogs through dissection of their esophageal nodules and the physical removal of the worms, without the necessity for collagenase digestion. Following nodule dissection, the harvested worms were deemed viable according to our established criteria of being pink in color, having an intact esophagus and showing some, albeit varied, motility. It would therefore appear that the physical harvesting of the worms had no adverse effect on parasite viability. A qualitative difference in movement was present between the worms, possibly due to the delay to time of nodule dissection and/or the pre-cooling that happens on placement of carcasses into a refrigerator at 4 °C, indicating that delays in harvesting should preferably be avoided.

Following harvest, the worms were placed in saline as the control or within Ham’s F12 medium, DMEM, RPMI 1640 medium and / or Iscove’s modification of DMEM for five to 7 days under serum free conditions. These media were selected based on prior reports of their use in in vitro culture of other parasites such as *Onchocerca* spp*.* and *Schistosoma* spp*.,* and these media are also the most commonly used media in mammalian cell culture. The media differed from each other in their amino acid and glucose content and in their buffering systems. For example, DMEM contained only sodium pyruvate and no buffer, RPMI 1640 medium contained only sodium bicarbonate as the buffer, Iscove’s modification of DMEM contained lower concentrations of bicarbonate as HEPES was included as the buffer and Ham’s F12 medium contained no buffer. In terms of their nutrient contents, the media contained different amino acids to support the growth of different cell types. Since *S. lupi* must survive off nutrients within their environment in esophageal nodules and with the selected cell media best representing the nutrient content within tissue fluid, it was speculated that these media would be able to meet the nutritional requirements of the parasite. Despite this, the parasites failed to thrive within the selected media with the worms progressively becoming weaker and more opaque until they died.

While we’re uncertain as to the exact reason for the earlier death of the parasites in the various media, this was most likely an indication of the parasite’s inability to utilise the nutrients within the media. This theory is supported by the measured change in pH of the media, as in all cases the parasites failed to modify the pH of the environment in which they were resident. From studies on *Ascaris suum* and *H. contortus*, one would have expected the parasite to modify their external environment to being a more acidic pH (circa pH 5 was reported for *A. suum*) [14, 15]. This pH modification is meant to arise from the excretion of the end-products of carbohydrate metabolism across the cuticle, in order to maintain the functionality of cuticle transporters. This would also explain why the parasites survived longer in the acidic saline, as the lower pH would have allowed for optimal functionality of the cuticle transporters for a longer period, with death probably eventually resulting from starvation as the incubation media was devoid of added nutrients. Support for the latter is evident by progressive increases in the parasite opaqueness, which from studies on *H. contortus* is an indication of glycogen stores depletion during starvation [16].

We propose some recommendations pertaining to future studies that potentially need the parasites to survive for a longer period of time. Firstly, the presence of *E. coli, E. faecalis, P. aeruginosa* and *S. marcescens*, indicates that the harvest of the parasites does result in the transfer of bacterial contaminants and that washing alone is not sufficient to remove them, warranting the need for antimicrobials in the media. Based on the organisms cultured, the aminoglycosides may a good antibiotic to include in the media. Nonetheless, we cannot at this stage associate the death of the parasite with the presence of the bacteria, as the four bacteria species were cultured equally well from all the media and the saline. The implication is that the bacteria cultured may be associated with the parasite, which is not uncommon as other nematode parasites have been reported to have bacteria attached to their cuticle e.g. urinary cysts caused by *Strongylus edentatus* are associated with genera such as *Escherichia*, *Enterobacter* and *Streptococcus* that are attached to the cuticle, while *E coli* and Pseudomonas have been associated with swine ascarids [17]. We also consider it unlikely that the bacteria were the cause of death of the parasites, especially since an intact cuticle purportedly protects nematode parasites against environmental insult [18]. Furthermore, living *S. lupi* parasites within esophageal nodules in infected dogs are routinely associated with purulent inflammation, necrotic cell debris and assorted bacteria in migratory tracts within these nodules. It may even be possible that the *S. lupi* parasite is the source of esophageal nodule contamination in a similar manner as described for *S. edentatus* cysts above.

A second consideration would be to lower the pH of the incubation media to 5 or 6, as the parasite may only be able to mobilise nutrients from the media at a lower pH, as seen with studies on Ascaris where it has been demonstrated that trans-cuticle transport is dependent on the pH of the surrounding environment [14]. Lastly perhaps the media should be supplemented with fetal calf serum (FCS). For this study, with the attempt being the characterization of excretory/secretory protein products, we kept the additions of exogenous proteins to the media to a minimum. This is in contrast with other studies for which fetal calf serum is commonly added to mammalian and parasite cell cultures as a source of albumin and growth factors. For the parasite *Schistosoma spp.,* it has been shown that the parasite needs albumin for glycogen production, while supplementation with 10% FCS was vital for the prolonged viability of *Oesophagostomum gutturosa* adult parasites cultured in vitro using RPMI 1640 medium, Iscove’s Modified Dulbecco’s Medium and Minimum Essential Medium for 39 days [17]. The FCS is believed to provide parasites with necessary growth factors, e.g. platelet-derived growth factor (PDGF), epidermal growth factor (EGF), transforming growth factor-^β^ (TGF-^β^) and insulin-like growth factor (IGF) [11].

## Conclusion

The aim of this study was to culture adult *S. lupi* parasites in vitro using serum-free media to harvest the ESPs, for subsequent carcinogenic studies. While we were able to keep the parasite alive ex vivo for a short period of time, we were unable to demonstrate carcinogenic effects in cultured fibroblasts. We did, however, confirm that the ESPs are composed of proteins.
